# Development and validation of an intra-tumor heterogeneity-related signature to predict prognosis of bladder cancer: a study based on single-cell RNA-seq

**DOI:** 10.18632/aging.203353

**Published:** 2021-08-02

**Authors:** Ranran Zhou, Jingjing Liang, Qi Chen, Hu Tian, Cheng Yang, Cundong Liu

**Affiliations:** 1 Department of Urology, The Third Affiliated Hospital of Southern Medical University, Guangzhou, China; 2 The Third School of Clinical Medicine, Southern Medical University, Guangzhou, China; 3 Department of Cardiology, Shunde Hospital of Southern Medical University, Foshan, China

**Keywords:** intra-tumor heterogeneity, bladder cancer, scRNA-seq, prognosis, risk model

## Abstract

Intra-tumor heterogeneity (ITH) was a potential mechanism of progression and drug resistance in bladder cancer (BCa). However, the understanding of ITH in BCa remains insufficient. Single-cell RNA sequencing (scRNA-seq) profiles of 2075 cells were analyzed, and 2940 cell markers were screened. The ITH of 396 cases was evaluated, and 96 ITH-related genes were identified. Based on the gene-pair strategy, 96 genes were cyclically paired, and an 8-gene-pair model was successfully established to evaluate the overall survival of BCa through Lasso and multivariate Cox regressions. The risk model showed high predictive value in the training dataset (*n* = 396, *p* = 0) and external validation datasets (*n* = 165, *p* = 2.497e-02; *n* = 224, *p* = 3.423e-02). The model was also valuable for the prediction of clinical treatment outcomes. Totally, a prognostic model based on scRNA-seq and ITH was successfully constructed and validated in large cohorts, providing novel clues for ITH study of BCa.

## INTRODUCTION

Bladder cancer (BCa), as the twelfth most common malignancies around the world, brings a tremendous social burden [1]. The 5-year survival rate of muscle-invasive bladder cancer (MIBC), one of the main subtypes of BCa, is dismal: 5%–30% [2]. Nowadays, some novel therapeutic methods, such as cisplatin-based neoadjuvant chemotherapy and immune checkpoint inhibitors (ICIs), have been proposed, making considerable strides in BCa treatment [3]. However, many BCa patients could not benefit from the current therapeutic regimens [4, 5]. Therefore, reliable prediction of prognosis was urgently demanded, which played an important role in guiding clinical treatment.

With the proliferation of tumor cells, the genomic characteristics of progeny cells are different from that of their parents, inducing the alternation of drug susceptibility, invasiveness, migration, and growth, which is known as intra-tumor heterogeneity (ITH) [6]. ITH is closely correlated with immunotherapy response because the neoantigen on tumor cells with high ITH is diluted, and the concentration is not enough to cause the antitumor immunity [7]. ITH is also capable of predicting the prognosis of patients with malignancies [8]. The underlying mechanisms of ITH include telomere damage, DNA mismatch repair deficiency, microsatellite instability (MSI), and epigenetic changes [9], but the understanding of ITH is far from enough for the moment.

Various methods, like fluid biopsy, gene sequencing, and multi-regional biopsy, have been developed to estimate the ITH of BCa patients. Sing-cell RNA sequencing (scRNA-seq) has attracted more and more attention due to its high resolution [10]. For instance, Maynard et al. disclosed the dynamic changes of lung tumor cells in patients who received target therapy via scRNA-seq technology [11], showing single-cell sequencing was a powerful tool for ITH research.

In the present study, we quantified the ITH of 396 cases with BCa from The Cancer Genome Atlas (TCGA). Then scRNA-seq data was collected from Gene Expression Omnibus (GEO), where we also downloaded the external validation datasets. A gene-pair strategy was implemented to improve the robustness of the established model [12]. The correlation between risk signature and clinical treatment outcomes was also explored. Our research constructed a promising tool to predict the clinical outcomes and provided some novel biomarkers, deepening the understanding of ITH in BCa.

## RESULTS

### ITH estimation of 396 cases with BCa

**Figure 1:**
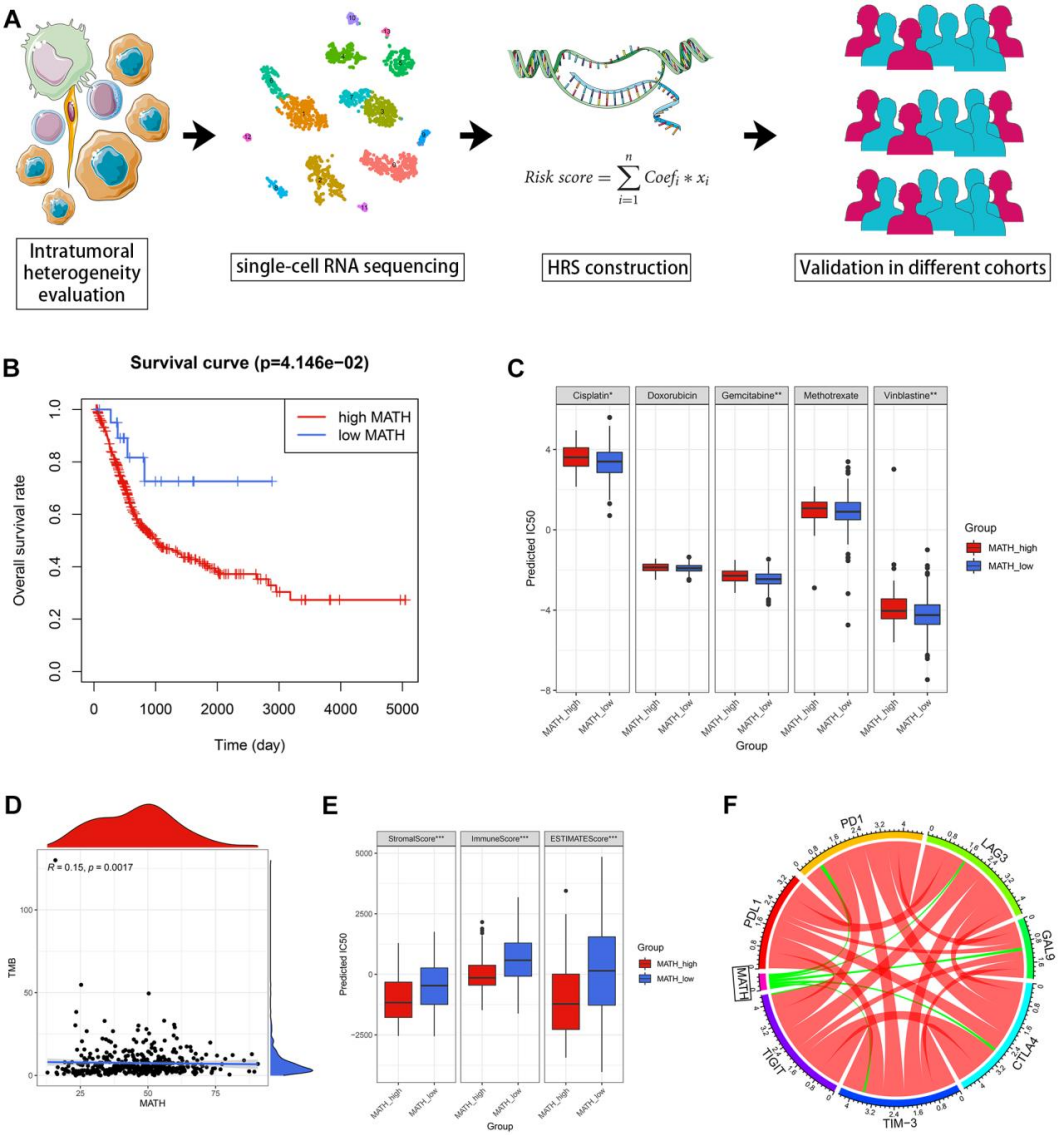
**Evaluation of ITH with MATH in BCa.** (**A**) The workflow of this study. (**B**) The patients with high MATH values suffered an unfavorable prognosis. (**C**) The estimated MATH values acted as a potential predictor for chemosensitivity with Wilcoxon signer-rank test. (**D**) MATH values were positively correlated with TMB. (**E**) The cases in the high-MATH group had significantly lower immune and stromal components in TME. (**F**) Spearman correlation analysis indicated MATH values were negatively correlated with routine immune checkpoint genes, including *PD1, LAG3, GAL9, CTLA4, TIM-3,* and *TIGIT.* The red lines and green lines represented positive correlation and negative correlation, respectively. The boldness of the lines was positively associated with the strength of the correlation. ^*^p < 0.05; ^**^p < 0.01; ^***^p < 0.001.

The workflow chart of this study is shown in Figure 1A. We utilized the mutant-allele tumor heterogeneity (MATH) algorithm to evaluate ITH of BCa cases from TCGA (Supplementary Table 1). Accordingly, the ITH of each individual was quantified as MATH value, and high MATH represented increased ITH of malignant tumors [13]. It was found that the BCa cases with high MATH suffered a poorer survival rate (*p* = 4.146e-02, Figure 1B) and lower sensitivity to chemotherapeutic agents (Figure 1C). The predictive potential of MATH to immunotherapy effectiveness was also evaluated. MATH was positively correlated with Tumor Mutational Burden (TMB) via Spearman correlation (r = 0.15, *p* = 0.0017, Figure 1D). With ESTIMATE algorithm, the proportion of immune and stromal components of the tumor microenvironment (TME) was calculated [14]. The ratios of immune and stromal components in TME in the low-MATH group were significantly higher than those in the high-MATH group by Wilcoxon test (*p* < 0.001, Figure 1E). The expression level of routine immune checkpoint genes, including *PD1, LAG-3, GAL-9, CTLA-4, TIM-3*, and *TIGIT*, were negatively correlated with MATH (*p* < 0.05, Figure 1F).

### Profiling of scRNA-seq data

**Figure 2:**
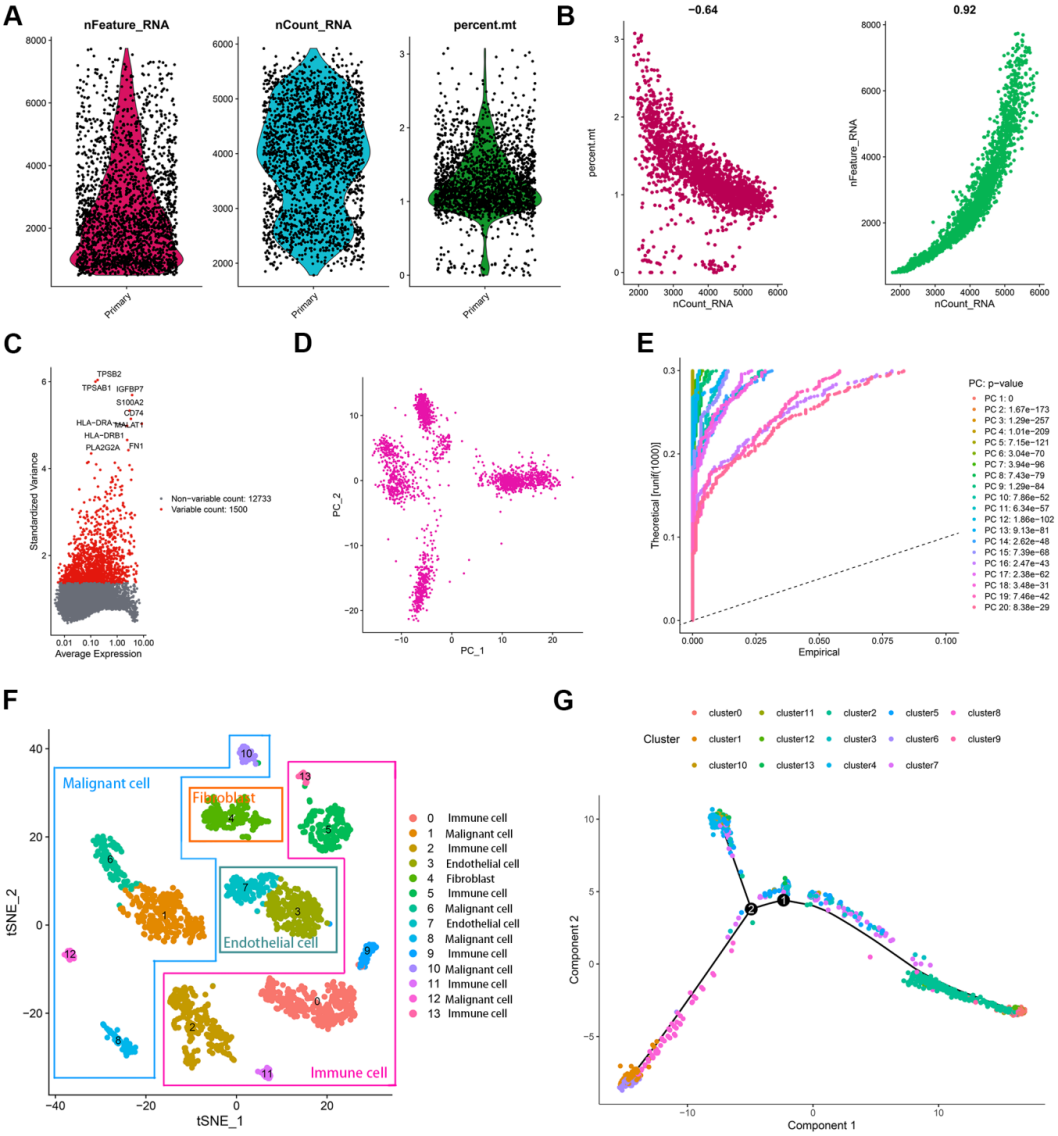
**Characterization of scRNA-seq from 2075 cells.** (**A**) Quality control plots of cell samples. (**B**) The sequencing depth was negatively correlated with the proportion of mitochondrial genes and positively associated with detected gene numbers. (**C**) 1500 variable genes across cell samples were identified. (**D**, **E**) PCA was conducted to reduce the dimension of data sets. (**F**) Cell samples were classified into 14 clusters with the t-SNE algorithm. (**G**) The trajectory analysis of 14 cell clusters.

The transcriptome sequencing of 2075 cells isolated from a patient with primary BCa was obtained from the GEO website [15]. Figure 2A displayed the detected gene numbers, sequencing count, and the percent of mitochondrial genes of each cell. With the sequencing depth increased, the percent of mitochondrial genes (r = –0.64) and detected gene count (r = 0.92) were also significantly changed (Figure 2B). The Top10 genes with the most significant variation across 2075 cells included *TPSB2, TPSAB1, IGFBP7, S100A2, CD74, HLA-DRA, MALAT1, HLA-DRB1, PLA2G2A,* and *FN1* (Figure 2C). The principal component analysis (PCA) was conducted to classify the cells preliminarily (Figure 2D). The *p*-value of each principal component (PC) was illustrated in Figure 2E, and the correlated genes of Top4 PC were shown in Supplementary Figure 1. To get a more precise clustering of cell samples, t-Distributed Stochastic Neighbor Embedding (t-SNE) was then implemented, and 2075 cell samples were divided into 14 different clusters (Supplementary Table 2, Figure 2F). A total of 2940 markers genes were screened with |logFC| > 0.5 and adjusted *p* < 0.05 (Supplementary Table 3), and accordingly, the cell types were annotated (Figure 2F). Figure 2G indicated the trajectory analysis of 14 cell clusters, which re-validated the annotation of cell types.

### ITH-related genes screening and heterogeneity-related score (HRS) construction

**Figure 3:**
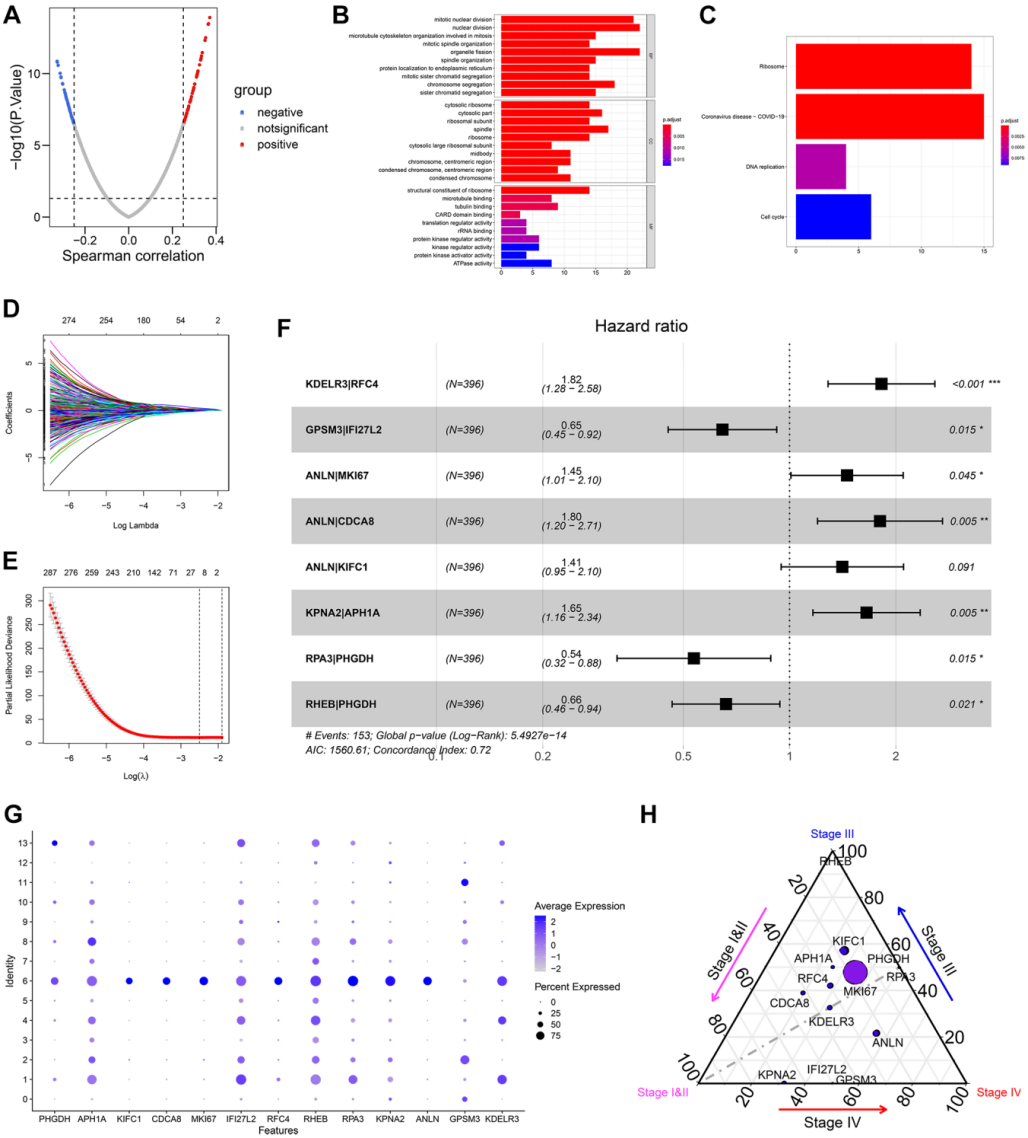
**Establishment of HRS.** (**A**) 96 of 2075 cell marker genes were significantly correlated with MATH. (**B**, **C**) GO functional annotation (**B**) and KEGG pathway enrichment (**C**) of the 96 genes. (**D**, **E**) 10 crucial gene-pairs correlated with OS were identified via Lasso-Cox regression. (**F**) 8 of 10 gene pairs were included in the prognostic model by multivariate Cox analysis with stepwise. (**G**) The expression values of 13 genes comprising the 8-gene-pair signature in 14 cell subpopulations. (**H**) The mutational rates of the 13 genes in different stages in BCa. The size of the bubble represented the mutational rates in all samples.

**Figure 4:**
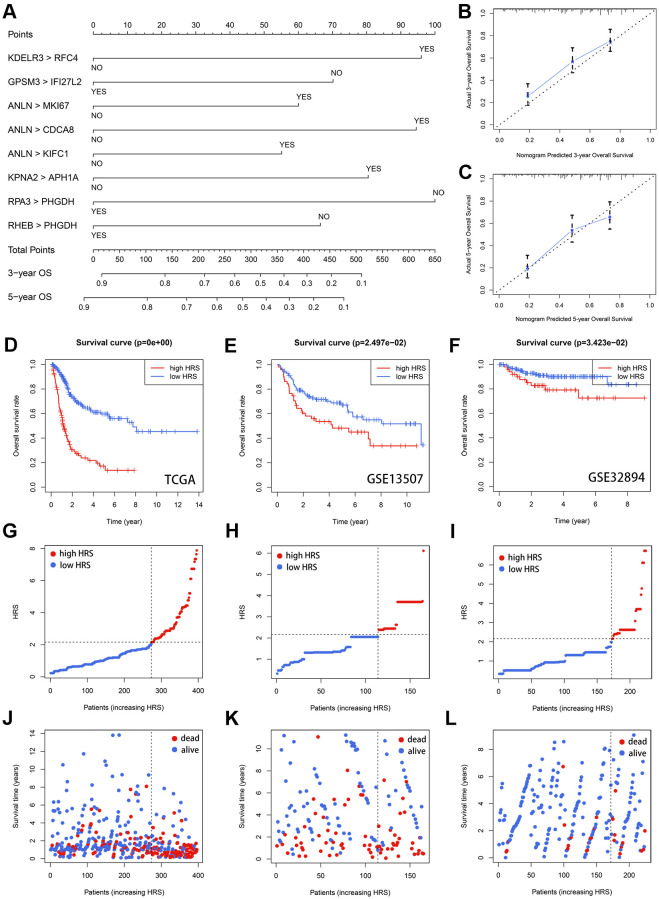
**Validation of HRS.** (**A**) A nomogram was plotted to visualize the HRS. (**B**, **C**) The calibration curves for 3- (**B**) and 5-year (**C**) OS prediction. (**D**–**F**) Kaplan-Meier survival analysis of HRS in TCGA (**D**), GSE13507 (**E**) and GSE32894 (**F**) cohorts. (**G**–**I**) The distribution of HRS in TCGA (**G**), GSE13507 (**H**) and GSE32894 (**I**). (**J**–**L**) The distribution of survival status in TCGA (**J**), GSE13507 (**K**) and GSE32894 (**L**).

We calculated the Spearman correlation coefficients between MATH values and transcriptome expression levels of 2940 cell markers, and 96 genes were screened (r > 0.25, *p* < 0.05, Supplementary Table 4, Figure 3A). Gene Ontology (GO) and Kyoto Encyclopedia of Genes and Genomes (KEGG) functional annotation indicated the 96 genes mainly were enriched in the biological process related to the cell cycle (Figure 3B and 3C). After cyclically pairing the screened 96 genes, 1256 gene pairs were identified. Subsequently, 10 ITH-related gene-pairs were extracted with the Lasso-Cox algorithm (Figure 3D and 3E), 8 of which were included in the prognostic model via multivariate Cox regression with stepwise (Supplementary Table 5, Figure 3F). Here, we defined the risk score, which was calculated based on the established risk model, as a heterogeneity-related score (HRS). To help clinicians better understand HRS, a nomogram was drawn (Figure 4A). The expression level of 13 genes, which comprised the 8-gene-pair model, in 14 different cell subgroups were sown in Figure 3G. Figure 3H illustrated the mutational rates of the genes in different tumor pathological stages.

### Validation of HRS

**Figure 5:**
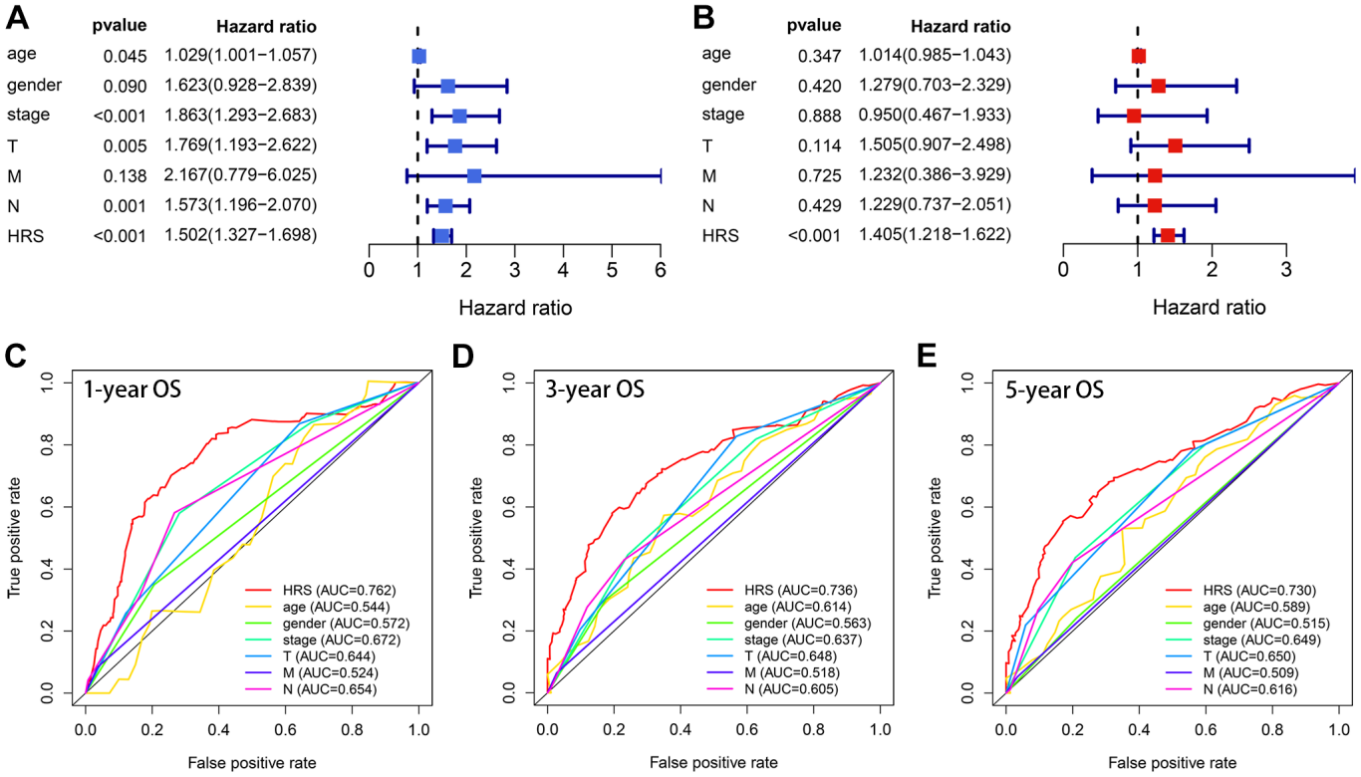
**HRS showed superiority in OS prediction compared with the clinicopathological features.** (**A**, **B**) HRS was an independent prognosis predictor in univariate (**A**) and multivariate (**B**) analyses. (**C**–**E**) ROC analysis indicated HRS had better ability than the clinical traits in 1- (**C**), 3- (**D**) and 5-year (**E**) OS prediction.

The calibration analysis indicated the estimated 3- (Figure 4B) and 5-year (Figure 4C) OS rates from HRS were close to the actual survival rates. The optimal HRS value, which was detected through X-tile software [16], was equal to 2.16, according to which each case was labeled with low- or high-risk (Supplementary Table 6). Compared with the patients with low-risk, the individuals with high-risk suffered significantly poorer prognosis (*p* = 0, Figure 4D). With HRS increasing, more deaths were observed (Figure 4G and 4J). Besides, HRS could be used to evaluate the survival rate in almost all subgroups by stratification survival analysis (Supplementary Figure 2). The external validation was also conducted in GSE13507 and GSE328094 datasets, which included 165 and 224 cases, respectively. The details of clinicopathological features of the datasets enrolled were s shown in Table 1. Based on the optimal cut-off of 2.16, the patients from these two researches were divided into low- and high-risk subgroups (Supplementary Tables 7 and 8). Kaplan-Meier survival plots displayed HRS could effectively predict the survival rates in GSE13507 (*p* = 2.497e-02, Figure 4E) and GSE32894 (*p* = 3.423-02, Figure 4F) cohorts. The distribution of HRS and survival status of GSE13507 (Figure 4H and 4K) and GSE32894 (Figure 4I and 4L) were also analyzed and illustrated. Wilcoxon signed-rank test conformed that the 13 genes involved in HRS were mostly differentially expressed between adjacent normal and BCa tissues (Supplementary Figure 3), and most of them could predict OS with significant efficacy (Supplementary Figures 4 and 5, Supplementary Tables 9–11). Besides, we also compared the mRNA expression level of the 13 genes between normal urothelium cell line SV-HUC-1 and BCa cell line T24 via Real-time quantitative PCR (RT-qPCR, Supplementary Figure 6), and the sequences of primers utilized are shown in Supplementary Table 12.

**Table 1:** The baseline information of 785 cases enrolled in the present study.

**Parameters**	**TCGA (*n* = 396)**	**GSE13507 (*n* = 165)**	**GSE32894 (*n* = 224)**
**Survival status**			
Alive	243 (61.3%)	96 (58.1%)	199 (88.8%)
Dead	153 (38.6%)	69 (41.8%)	25 (11.1%)
Follow-up (day)	778.19 ± 814.38	1451.45 ± 1127.70	1196.98 ± 767.38
Age	67.84 ± 10.53	65.18 ± 11.93	69.43 ± 11.28
**Gender**			
Female	104 (26.2%)	30 (18.1%)	61 (27.2%)
Male	292 (73.7%)	135 (81.8%)	163 (72.7%)
**Pathological Stage**			
I	2 (0.5%)	–	–
II	124 (31.3%)	–	–
III	138 (34.8%)	–	–
IV	130 (32.8%)	–	–
Unknown	2 (0.5%)	–	–
**pT stage**			
T0	1 (0.2%)	0 (0.0%)	0 (0.0%)
Ta	0 (0.0%)	23 (13.9%)	110 (49.1%)
T1	3 (0.7%)	81 (49.0%)	63 (28.1%)
T2	113 (28.5%)	31 (18.7%)	43 (19.1%)
T3	190 (47.9%)	19 (11.5%)	7 (3.1%)
T4	57 (14.3%)	11 (6.6%)	1 (0.4%)
Unknown	32 (8.0%)	0 (0.0%)	0 (0.0%)
**M stage**			
M0	189 (47.7%)	158 (95.7%)	–
M1	10 (2.5%)	7 (4.2%)	–
Unknown	197 (49.7%)	0 (0.0%)	–
**pN stage**			
N0	229 (57.8%)	149 (90.3%)	27 (12.0%)
N1	44 (11.1%)	8 (4.8%)	3 (1.3%)
N2	75 (18.9%)	6 (3.6%)	10 (4.4%)
N3	7 (1.7%)	1 (0.6%)	0 (0.0%)
Unknown	41 (10.3%)	1 (0.6%)	184 (82.1%)
**Risk stratification**			
High	123 (31.0%)	51 (30.9%)	52 (23.2%)
Low	273 (68.9%)	114 (69.0%)	172 (76.7%)
HRS	1.90 ± 1.58	1.96 ± 1.03	1.52 ± 1.20

Abbreviations: TCGA: The Cancer Genome Atlas; HRS: heterogeneity-related score.

### Clinical evaluation by HRS

**Figure 6:**
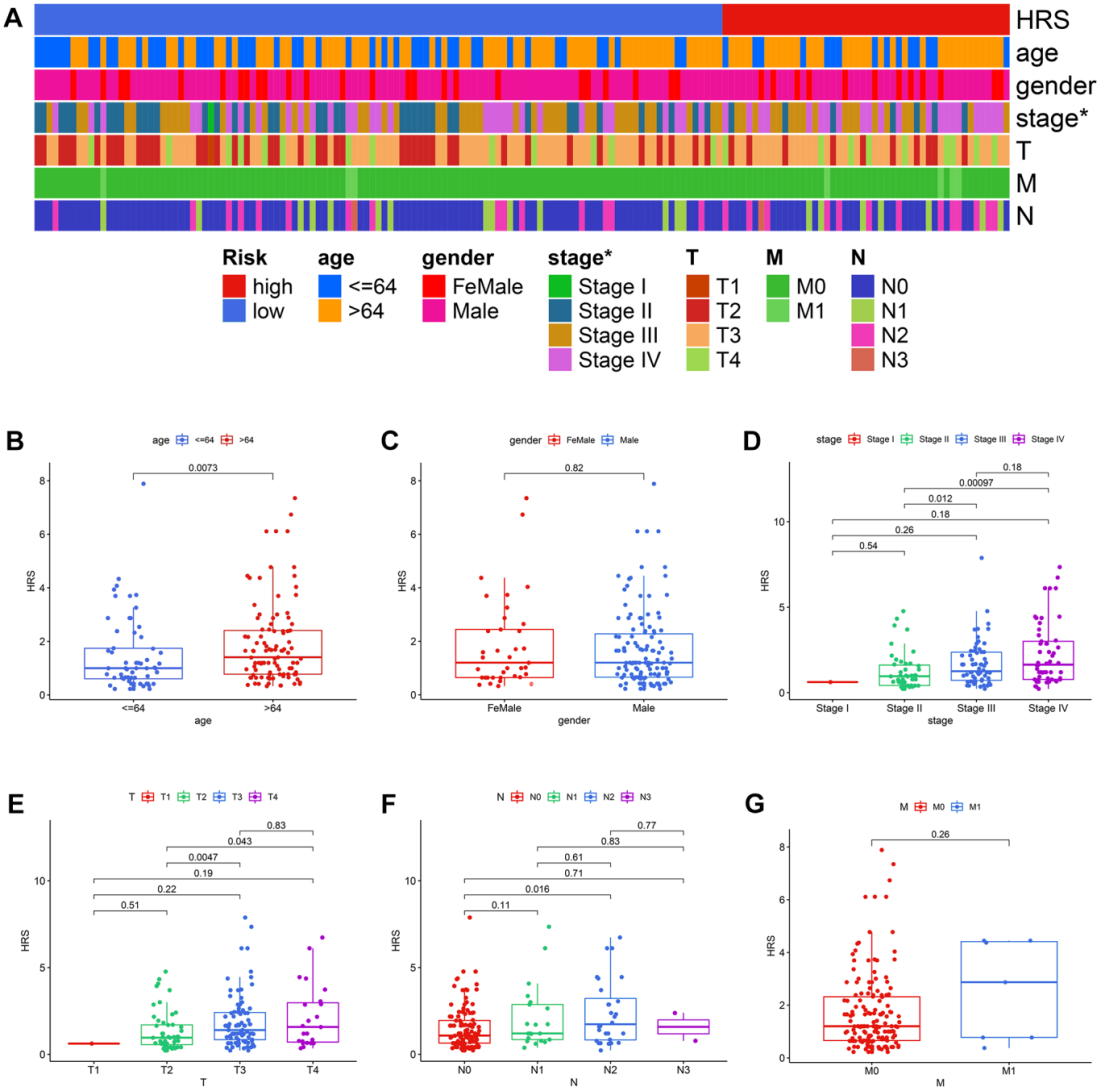
**The correlation between HRS and other clinicopathological variables.** (**A**) The strip curve displayed HRS was significantly correlated with pathological tumor stages. (**B**–**G**) Correlation analysis of HRS with age (**B**), gender (**C**), pathological stages (**D**), pathological T stages (**E**), pathological N stages (**F**), and M stages (**G**).

Compared with other clinicopathological features, like age, gender, pathological stages, pathological T stages, M stages, and pathological N stages, HRS was an independent prognostic factor no matter in the univariate Cox (Figure 5A) and multivariate Cox (Figure 5B) analysis. The areas under curves (AUCs) of HRS were also higher than those clinical parameters in 1-year (Figure 5C), 3-year (Figure 5D) and 5-year (Figure 5E) receiver operating characteristic (ROC) curves. The strip curve indicated HRS was significantly associated with pathological tumor stages via the Chi-square test (*p* < 0.05, Figure 6A). Wilcoxon test displayed that the HRS was also correlated with other clinicopathological traits (Figure 6B–6G), which were widely regarded as risk factors for BCa.

### Correlation between HRS and clinical treatment

**Figure 7:**
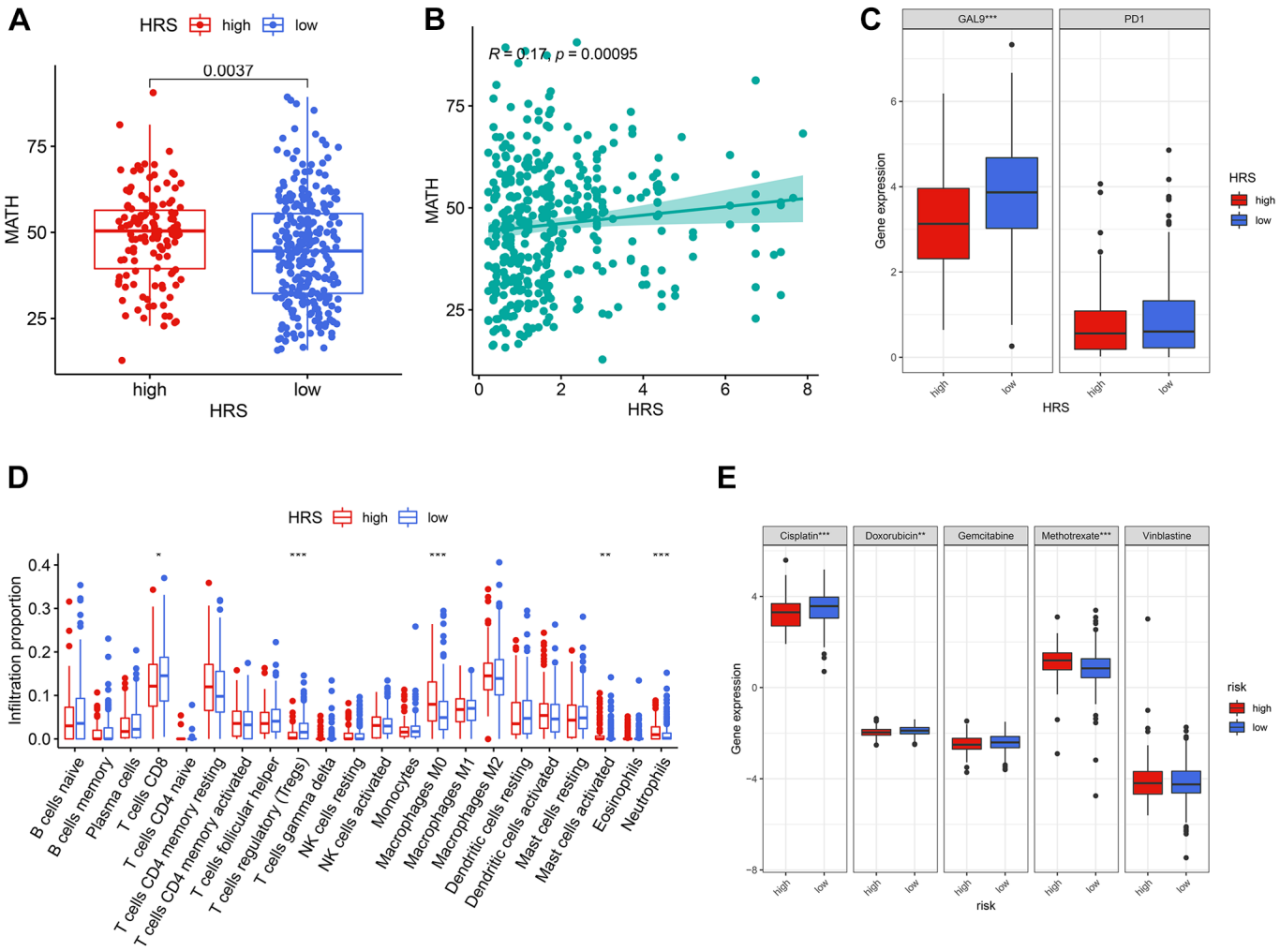
**HRS was a potential predictor for clinical treatment of BCa.** (**A**, **B**) HRS was significantly correlated with MATH, codetermined by difference (**A**) and correlation (**B**) analysis. (**C**) GAL-9 was differentially expressed between low- and high-HRS groups. (**D**) The cases in the high-HRS group were more likely to be associated with the high infiltration of M0 macrophages, activated mast cells, and neutrophils, whereas they were negatively correlated with the infiltration of CD8^+^ T cells and Tregs. (**E**) High HRS was linked to a lower IC50 for chemotherapeutics like cisplatin and doxorubicin, whereas it was correlated to a higher IC50 of methotrexate. ^*^p < 0.05; ^**^p < 0.01; ^***^p < 0.001.

HRS was significantly associated with MATH values through Wilcoxon signed-rank test (*p* = 0.0037, Figure 7A) and Spearman correlation analysis (r = 0.17, *p* = 0.00095, Figure 7B). We also found that the high-HRS group had a relatively lower *GAL-9* expression level than the low-HRS group via the Wilcoxon test (*p* < 0.001, Figure 7C). With the CIBERSORT algorithm [17], we estimated the infiltration proportion of 22 immune cells in TME and found some cell types, including CD8^+^ T cells, Tregs, M0 macrophages, resting mast cells, and neutrophils, were closely associated with HRS by Wilcoxon test (*p* < 0.05, Figure 7D). Most of the HRS-related immune cells have been reported to influence immunotherapeutic outcomes [18–20]. It was also found that the evaluated chemotherapy sensitivity to cisplatin, methotrexate, and vinblastine was different in the low-HRS and high-HRS subgroup by means of the Wilcoxon test (*p* < 0.05, Figure 7E). In all, HRS had the potential to serve as a biomarker for the effectiveness of clinical treatment, containing immunotherapy and chemotherapy, in BCa.

### HRS-related biological pathways detection

**Figure 8:**
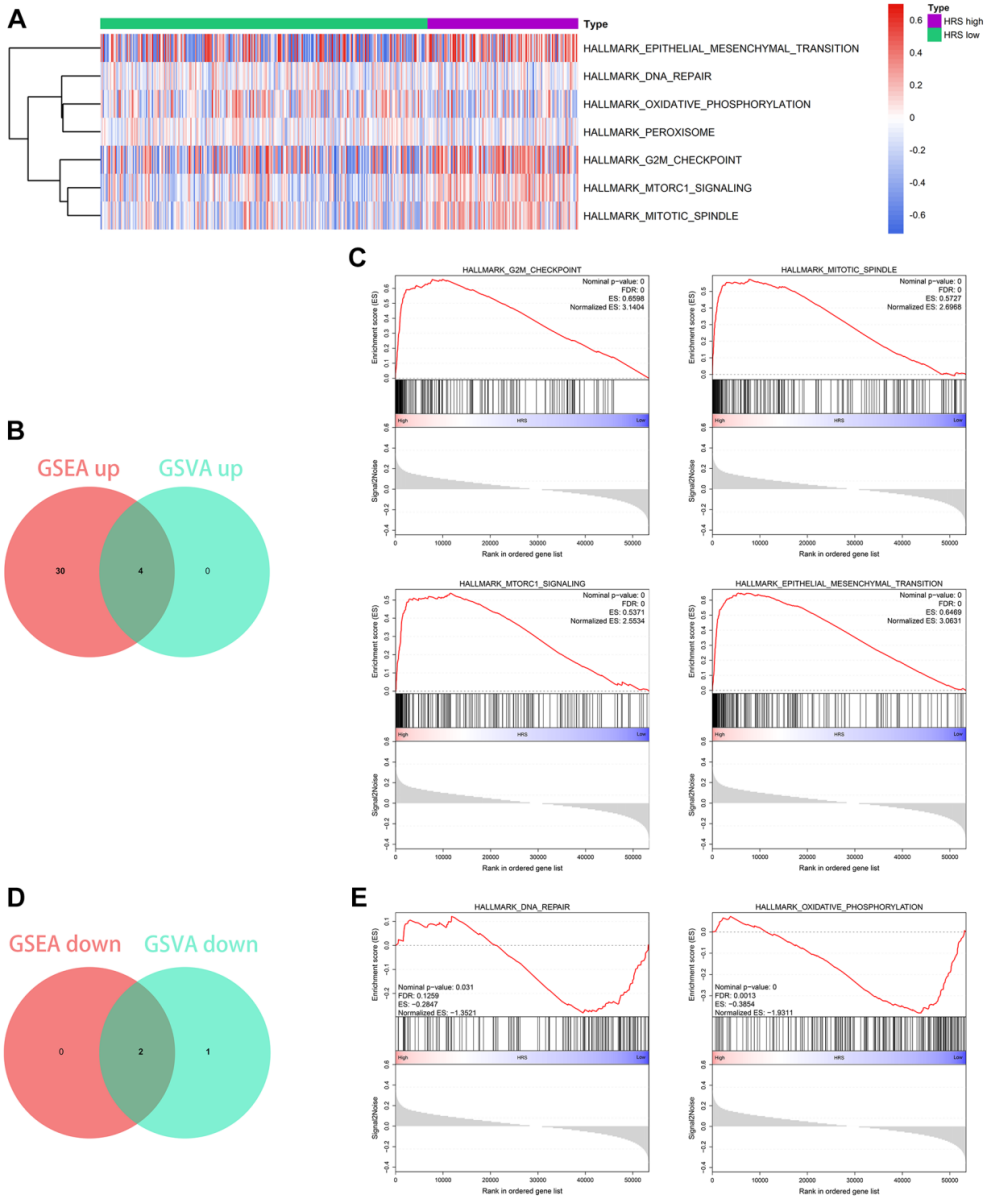
**Functional enrichment analysis.** (**A**) The heatmap showing the analysis results of GSVA. (**B**, **C**) Four pathways, including G2M checkpoint, mitotic spindle, mTORC1 signaling, and epithelial mesenchymal transition, were positively related to HRS, which was codetermined by GSEA and GSVA. (**D**, **E**) Two pathways, including DNA repair and oxidative phosphorylation, were negatively associated with HRS through the combined analysis by GSEA and GSVA.

Gene Set Enrichment Analysis (GSEA) and Gene Set Variation Analysis (GSVA) were both performed to ensure the predictive accuracy of the pathway enrichment results. The analysis results of GSVA were shown in Figure 8A and Supplementary Table 13, and GSEA results were supplemented in Supplementary Tables 14 and 15. Among the related biological process, G2M checkpoint, mitotic spindle, mTORC1 signaling, and epithelial-mesenchymal transition (EMT) were observed to be positively correlated with HRS (Figure 8B and 8C), while DNA repair and oxidative phosphorylation (OXPHOS) had a significantly negative association (Figure 8D and 8E).

### The candidate compounds targeting HRS

**Figure 9:**
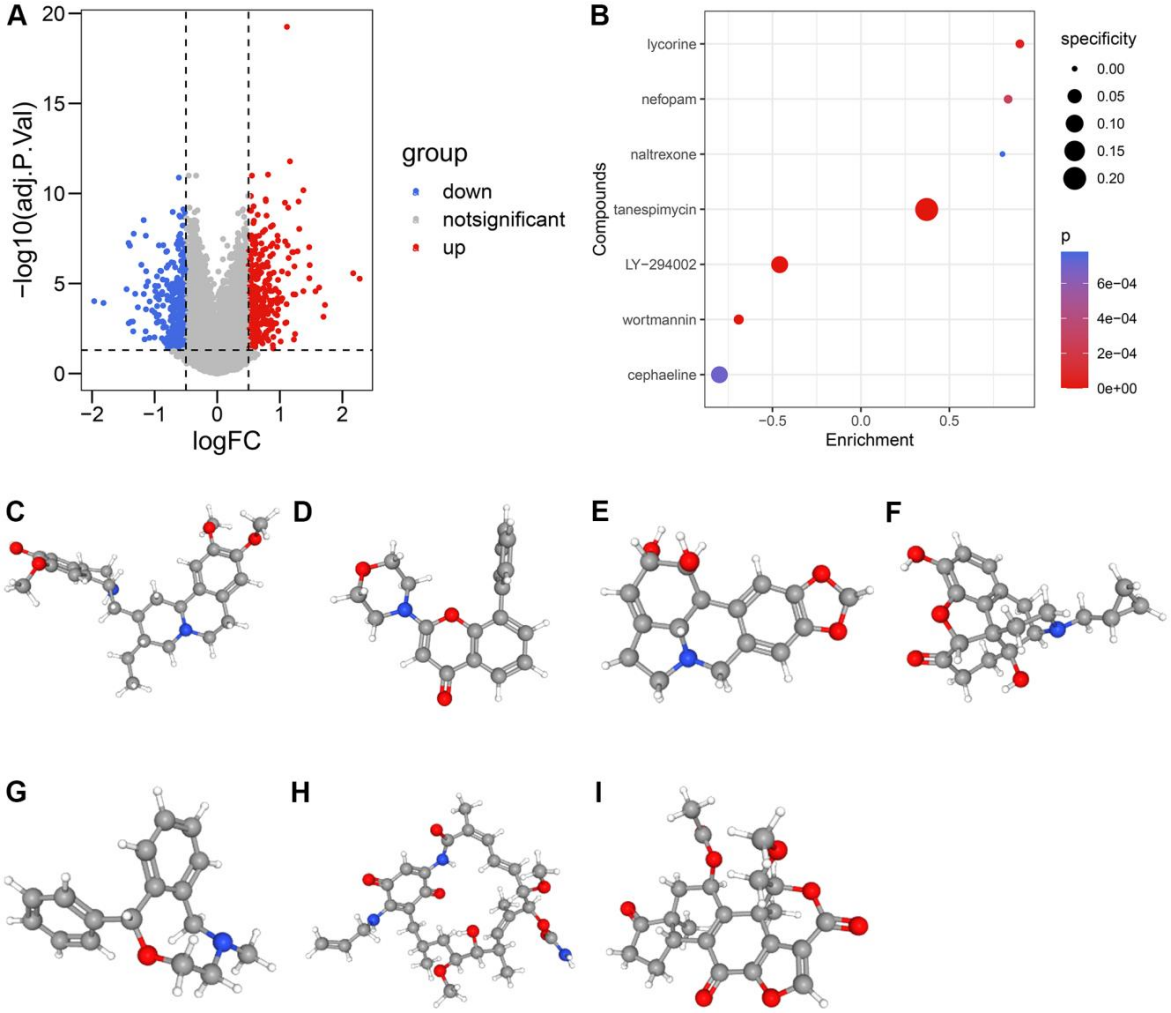
**The candidate compounds targeting HRS.** (**A**) The volcano plot displayed the DEGs between low-HRS and high-HRS cases. (**B**–**I**) Seven compounds, including cephaeline (**C**), LY-294002 (**D**), lycorine (**E**), naltrexone (**F**), nefopam (**G**), tanespimycin (**H**), and wortmannin (**I**), were identified.

To analyze the potential HRS-related compounds, we uploaded the differentially-expressed genes (DEGs) extracted from high-HRS and low-HRS patients and illustrated in Figure 9A and Supplementary Table 16 to the CMap database. Seven potential compounds, including cephaeline (Figure 9C), LY-294002 (Figure 9D), lycorine (Figure 9E), naltrexone (Figure 9F), nefopam (Figure 9G), tanespimycin (Figure 9H), and wortmannin (Figure 9I), were the predicted small molecule drugs with the most significance (Figure 9B, Supplementary Table 17).

## DISCUSSION

BCa is among the most malignant tumors around the world. The prognosis of BCa is unfavorable, especially for the cases with locally advanced cancers and distant metastasis [21]. Therefore, the accurate prediction of BCa prognosis remains one of the topics we are concerned about most. ITH, a hallmark of malignant cancers, refers to the change of molecular biology and genomic features in tumor cell evolution [22]. It has been widely accepted that ITH acted as one of the fundamental causes of many phenotypes of cancers. Recent research characterized ITH across 38 different tumors, uncovering some latent driving factors [9]. Nevertheless, it is a long and complex process to comprehensively understand ITH, and more studies should be conducted. At present, more and more evolution traits of malignant cells have been revealed based on single-cell level [23], demonstrating single-cell technology was a valuable maneuver to analyze ITH.

Some previous studies contributed to the prognosis prediction of BCa. For example, according to TP53 mutation status, Wu et al. developed and validated a predictive model for OS of BCa [24]. Based on glucose metabolism, an 18-gene signature was successfully built to predict prognosis in urothelial carcinoma [25]. These efforts helped for individualized treatment and the underlying mechanisms exploring. However, the prognostic value of ITH-related signature has not been reported in BCa. Besides, most of the previous studies were based on the DEGs identified from the tissues with different statuses, like tumor and paracarcinoma, or high and low immune infiltration, which might omit important biomarkers because of the ignorance of the differences among various cell subsets. Given the reason mentioned above, some researchers have constructed and successfully validated the scRNA-seq-based prognostic models in cancers [26, 27], but no risk signature on the basis of scRNA-seq has developed in BCa. Therefore, the present study established an ITH-related prognosis signature based on scRNA-seq, which was reasonable and urgently demanded.

This study first evaluated the ITH of 396 BCa cases from the TCGA-BLCA dataset via the MATH algorithm, which was developed based on mutant-allele fractions (MAFs) and validated in head and neck squamous cell carcinoma [8, 13]. The MATH values were positively correlated with ITH, as well as the unfavorable survival rate (Figure 1B). Then scRNA-seq data of 2075 cells obtained from a patient with primary BCa was analyzed, and 14 different cell types were identified. Based on the cell markers of cell clusters, 96 genes were screened as ITH-related genes via co-expression analysis with MATH values. GO and KEGG analysis displayed that the genes mainly were enriched in cell cycle-related pathways. Subsequently, 96 genes were cyclically paired, and a 0-or-1 matrix was built with gene-pair strategy, where there was no need for definite expression level of genes. Lasso and multivariate Cox with stepwise helped establish an 8-gene-pair prognostic model, and accordingly, the heterogeneity-related score (HRS) was calculated for each individual. The cases with high HRS suffered poorer survival in the TCGA-BLCA, GSE13507, and GSE32895 datasets, indicating that HRS might serve as a reliable risk predictor. Besides, the expression value of *GAL-9* and CD8^+^ T cell infiltration proportion in the high-HRS group was significantly lower than that in the low-HRS populations, showing HRS might be linked to immunotherapy response. It was also found the HSR had the potential to indicate chemotherapy effectiveness, including cisplatin, doxorubicin and methotrexate.

Several biomarkers were firstly reported to be correlated with the development of BCa. *KDELR3*, which was up-regulated in tumor samples and cell lines compared with that in adjacent normal tissues and normal cell lines (Supplementary Figures 3 and 6), served as a predictor for poor prognosis of BCa (Supplementary Figures 4 and 5). It was reported that the loss of *KDELR3* in the genetically engineered mouse would lead to the metastasis depression of melanoma cells by interacting with KAI1, which was a metastasis suppressor [28]. *GPSM3* was found to be up-regulated in normal samples and the cases with favorable prognosis (Supplementary Figures 3–6), indicating *GPSM3* acted as a tumor suppressor for BCa. *GPSM3* was found to be a regulator to immune cells like leukocytes and monocytes [29, 30]. However, the functions of *GPSM3* in malignant tumors remain unclear. Other novel biomarkers, like *RFC4, RPA3, IFI27L2,* and *APH1A*, were also identified, despite the unclear associated mechanisms. Totally, our risk signature helped identify novel biomarkers and might provide clues for mechanisms of BCa from the prospect of ITH.

The potential compounds targeting HRS were also predicted with CMap. Some of the compounds could interact with malignant tumors from previous research. Lycorine promoted apoptosis of gastric tumor cells by FBXW7-MCL1 axis [31]. Low-dose naltrexone inhibited PI3K/AKT/mTOR pathway and thus suppressed the proliferation of cervical cancer cells [32]. Tanespimycin acted as an antineoplastic drug through targeting HSP90 [33]. Wortmannin was a DNA repair inhibitor and could delay the production of cisplatin resistance [34] in ovarian cancer. Here, we listed some potential compounds targeting ITH, which might help develop some new therapeutic plans.

The shortcomings of the present study should not be neglected. First, although the prognostic value of HRS was validated in 785 BCa cases from three different cohorts, a prospective, large-scale, and multi-center clinical trait would help revalidate the clinical usefulness. Second, several biological pathways and candidate compounds were found to be linked to HRS, which needed to be further experimental validated.

In a word, we developed a novel ITH-related signature to predict the prognosis of BCa based on scRNA-seq, and validated the robustness in large cohorts, which provided a promising tool for clinicians and identified novel biomarkers to disclose the underlying mechanisms.

## MATERIALS AND METHODS

### Transcriptome data acquisition

The RNA-seq data of 427 samples, containing 19 paracancerous and 408 BCa samples, were downloaded from the TCGA website (https://portal.gdc.cancer.gov/), along with the following-up and clinicopathological information. The external validation datasets, which included GSE13507 and GSE32894, were obtained on the GEO database (https://www.ncbi.nlm.nih.gov/geo/), and their expression matrices were directly downloaded. After excluding the cases with the following-up less than 30 days, a sum of 785 cases, including 396 cases from TCGA, 165 patients from GSE13507 and 224 individuals from GSE32894, were enrolled in this study.

### ITH evaluation and TMB calculation

Here, we utilized a reported method, known as the MATH algorithm, to infer the degree of ITH [13, 35]. After obtaining the maf files in the TCGA-BLCA dataset with the TCGAmutations R package, we estimated the MATH value of each individual using the maftools package, which was also implemented to calculate the TMB based on the masked somatic mutation data downloaded from TCGA.

### Profiling of scRNA-seq

Compared with the DEGs screened from bulk-seq data, those extracted based on scRNA-seq data might be able to reflect the ITH with higher efficacy. Here, we downloaded the expression matrix of 2075 cells isolated from a patient with primary BCa from GEO database [15]. To make sure the DEGs were all obtained from human, we discarded two other samples in the raw dataset obtained from mice. Seurat package was used to filter the cells with poor quality, where the detected gene, cell, and mitochondria gene counts were also calculated. The cells with less than 200 genes detected and more than 5% of mitochondria gene proportion would be excluded. Based on the Top 1500 variable genes across all cell samples, PCA and t-SNE were then performed to classify the cell samples, and the marker genes were screened with |logFC| > 0.5 and adjusted *p* < 0.05 filtering. We annotated the cell categories based on the marker genes with CellMarker [36] (http://biocc.hrbmu.edu.cn/CellMarker/) and CancerSEA [37] (http://biocc.hrbmu.edu.cn/CancerSEA/) databases. Pseudotime analysis was conducted with the monocle package of R.

### Survival analysis

Lasso-Cox regression with 10-fold cross-validation and multivariate Cox analysis with stepwise were performed via glmnet and survival packages, respectively. The nomogram and calibration plots were drawn with rms package. Kaplan-Meier survival analysis with log-rank test was carried out with survival R package, where the optimal cut-off value was determined by X-tile [16]. SurvivalROC package helped to conduct the time-dependent ROC analysis.

### Gene-pair strategy

To ensure the risk model could suit the BCa samples tested by whatever technical means, a gene-pair strategy was adopted, where there was no need for concrete gene expression values [38]. If the mRNA expression value of gene A is higher than that of gene B, A plus B, or “A|B”, is defined as 0; otherwise, it will be regarded as 1. All genes will be cyclically paired, and a 0-or-1 matrix will be successfully constructed. Besides, if 0 or 1 accounts for less than 20% of the gene pairs, this pair would be considered to be meaningless and excluded from the present study.

### Evaluation of immune infiltration

To estimate the proportion of immune and stromal components in TME, we implemented the ESTIMATE algorithm [14], which has been widely utilized in many different tumors, including BCa [39]. Besides, the CIBERSORT algorithm was also used for the evaluation of the infiltration level of 22 immune cells [17].

### Chemotherapeutic sensitivity estimation

The half inhibitory centration (IC50) of the common chemotherapeutic drugs, like cisplatin, doxorubicin, gemcitabine, methotrexate, and vinblastine, were evaluated with the pRRophetic package on the basis of the transcriptome RNA-seq data.

### Functional enrichment analysis

GO and KEGG enrichment was performed through the clusterProfiler package. GSVA was conducted via GSVA package, and limma package was used for the HRS-related pathways screening under |logFC| > 0.05 and adjusted *p* < 0.05 threshold. GSEA software (version 4.1.0) was downloaded from GSEA’s official website (https://www.gsea-msigdb.org/gsea/), and Nom *p* < 0.05 and FDR q < 0.25 were set as the filtering criteria. The hallmark gene signature (version 7.2), downloaded from the Molecular Signatures Database (MSigDB), was chosen as the reference gene set.

### Identification of novel candidate compounds

Based on the transcriptome expression data of 396 cases from TCGA, the DEGs extracted between high-HRS and low-HRS groups were screened via limma package. The genes with |logFC| > 0.5 and adjusted *p* < 0.05 were considered to be significant. Subsequently, the gene symbols of the DEGs were all transformed into Affymetrix probe ID, which would be uploaded to the CMap website (https://portals.broadinstitute.org/cmap/). The predicted compounds with *p* < 0.001 would be considered meaningful, and the analysis results were visualized by the ggplot2 package. The 3D structures of the compounds were obtained from the PubChem database (https://pubchem.ncbi.nlm.nih.gov/).

### Cell culture and real-time quantitative PCR

T24 and SV-HUC-1 cell lines were purchased from the Chinese Academy of Sciences Shanghai Cell Bank, and cultured in McCoy’s 5A modified medium (Gibco, USA) and F12K medium (Gibco, USA), respectively. 1% antibiotic and 10% fetal bovine serum (FBS, Gibco, USA) were added to the medium. The cells were maintained in a humidified atmosphere with 5% CO2 at 37°C.

According to the manufacturer’s protocol, the total RNA of these cells was extracted with Trizol (ThermoFisher Scientific, Germany). First-strand cDNA was synthesized with PrimeScript RT Reagent Kit (Takara, China) and amplified by SYBR Premix ExTaq kit (Takara, China). The mRNA expression values were detected via ABI Prism 7000 (Applied Biosystems, USA). GAPDH was chosen as an internal reference, and 2^−ΔΔC^ was utilized to calculate the gene expression values. Student’s *t* test was used to detect the expression difference. All the primers were obtained from PrimerBank (https://pga.mgh.harvard.edu/primerbank/) and shown in Supplementary Table 12.

## Supplementary Materials

Supplementary Figures

Supplementary Table 1

Supplementary Table 2

Supplementary Table 3

Supplementary Table 4

Supplementary Tables 5, 9-13

Supplementary Table 6

Supplementary Table 7

Supplementary Table 8

Supplementary Table 14

Supplementary Table 15

Supplementary Table 16

Supplementary Table 17
