# Supplementary material for: Development and validation of an intra-tumor heterogeneity-related signature to predict prognosis of bladder cancer: a study based on single-cell RNA-seq
Source: Aging (Albany NY). 2021 Aug 2;13(15):19415–41. doi: 10.18632/aging.203353 (PMC8386527; doi:10.18632/aging.203353)
Supplement: Supplementary Figures [file aging-13-203353-s001.pdf]

SUPPLEMENTARY FIGURES

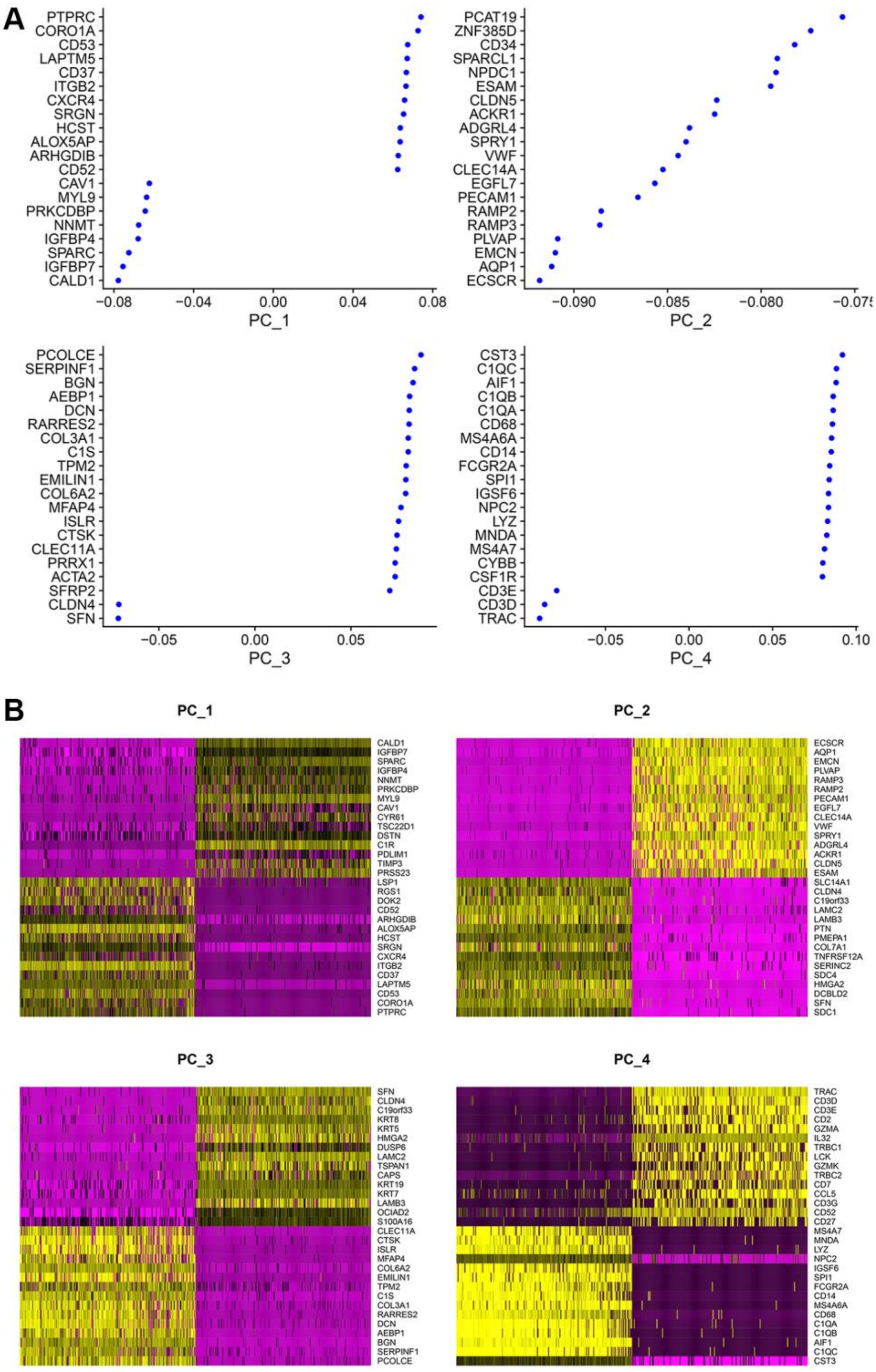

Supplementary Figure 1. The Top 4 components and the correlated genes in PCA analysis. (**A**) The Top related genes to each principal component. (**B**) The heatmap indicating the expression level of the Top related genes. The colors ranging from purple to yellow represented the expression values from low to high.

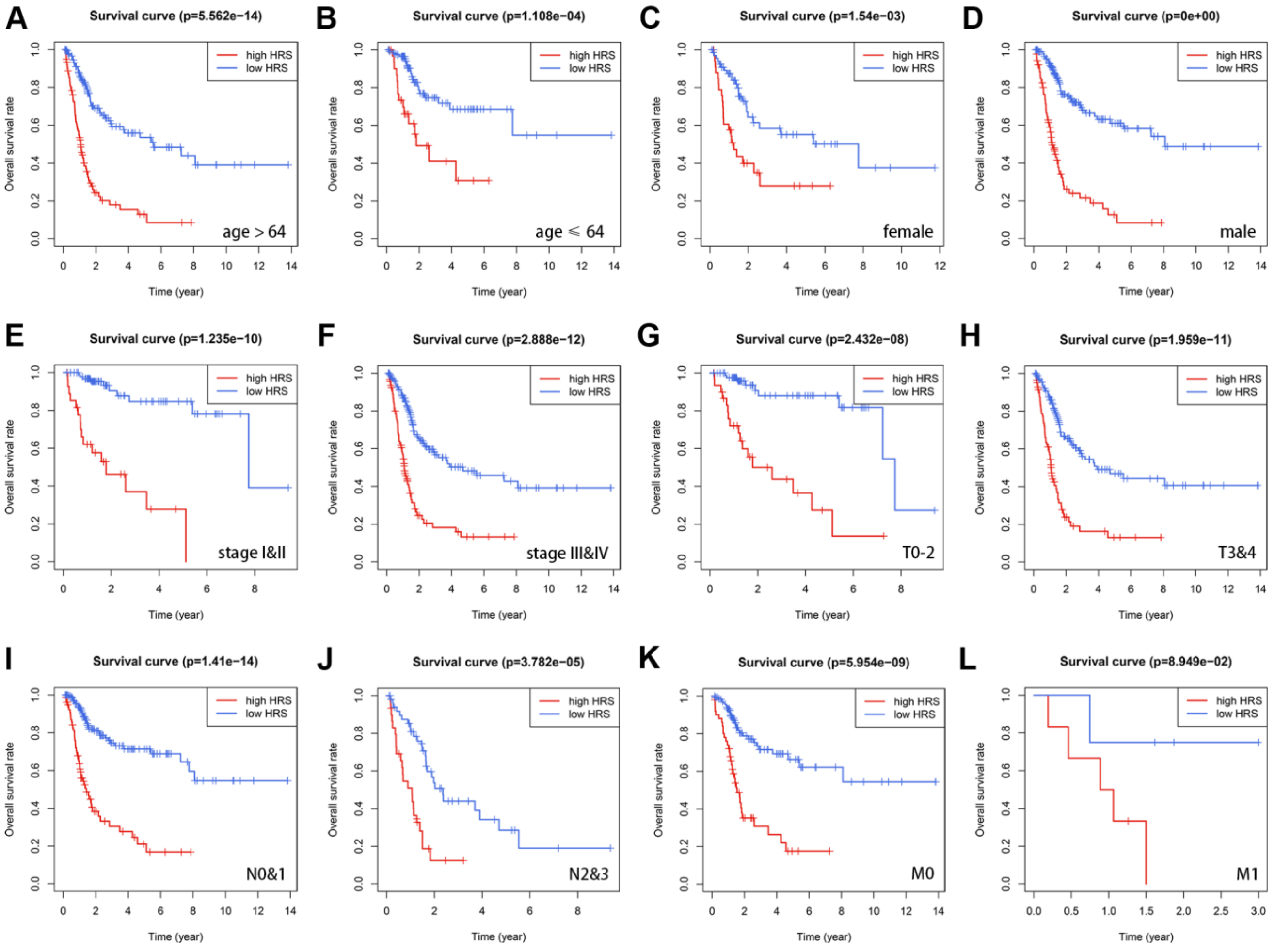

Supplementary Figure 2. The Kaplan-Meier survival stratification analysis in TCGA-BLCA cohorts. (**A**) Age > 64 years. (**B**) Age ≤ 64 years. (**C**) Female. (**D**) Male. (**E**) Pathological stage I and II. (**F**) Pathological stage III and IV. (**G**) Pathological T0-2. (**H**) Pathological T3 and 4. (**I**) Pathological N0 and 1. (**J**) Pathological N2 and 3. (**K**) M0. (**L**) M1.

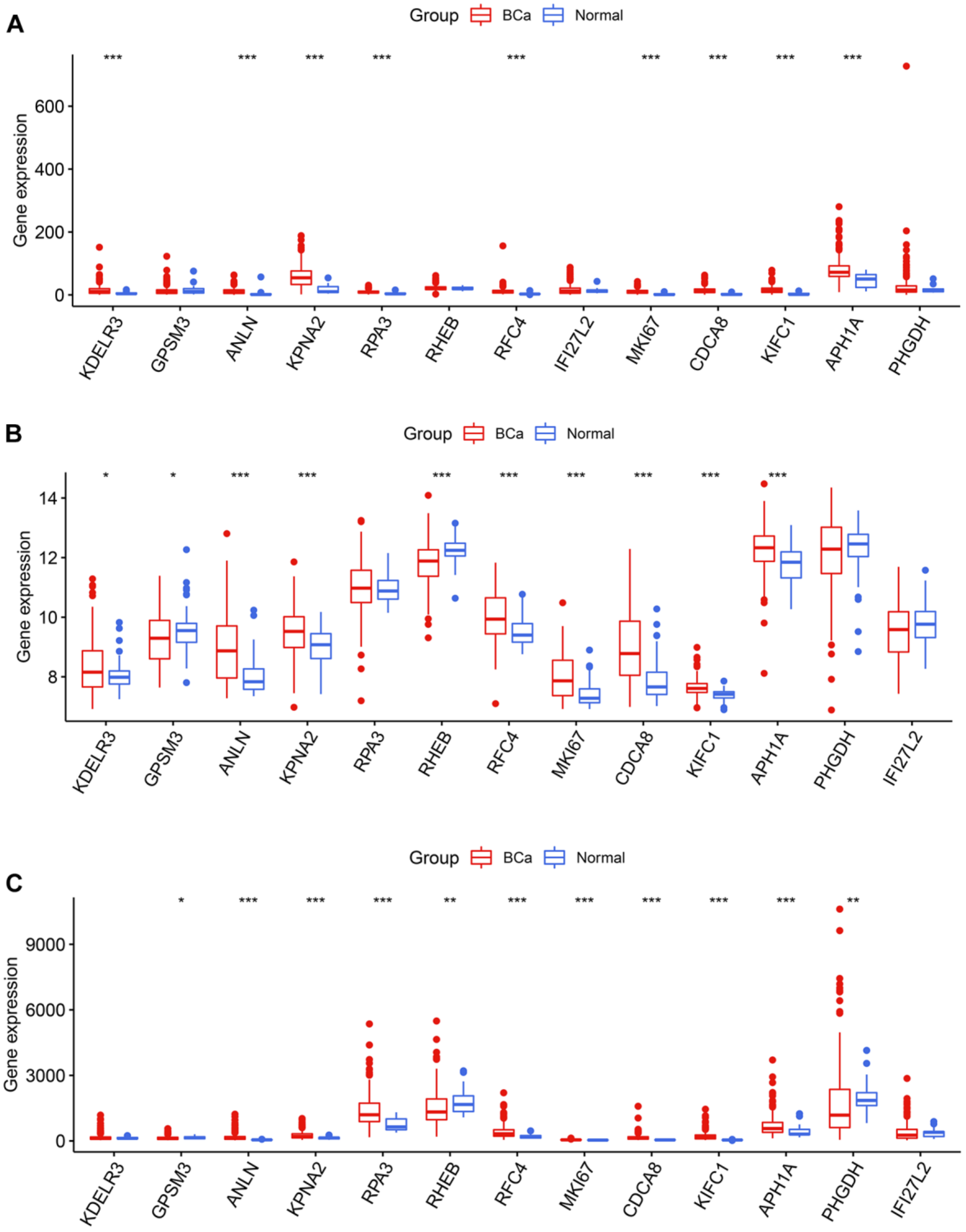

Supplementary Figure 3. The expression level of 13 genes comprising HRS in paracancerous and BCa tissues in TCGA (**A**), GSE13507 (**B**), and GSE32894 (**C**) cohorts. $^{*}p < 0.05$; $^{**}p < 0.01$; $^{***}p < 0.001$.

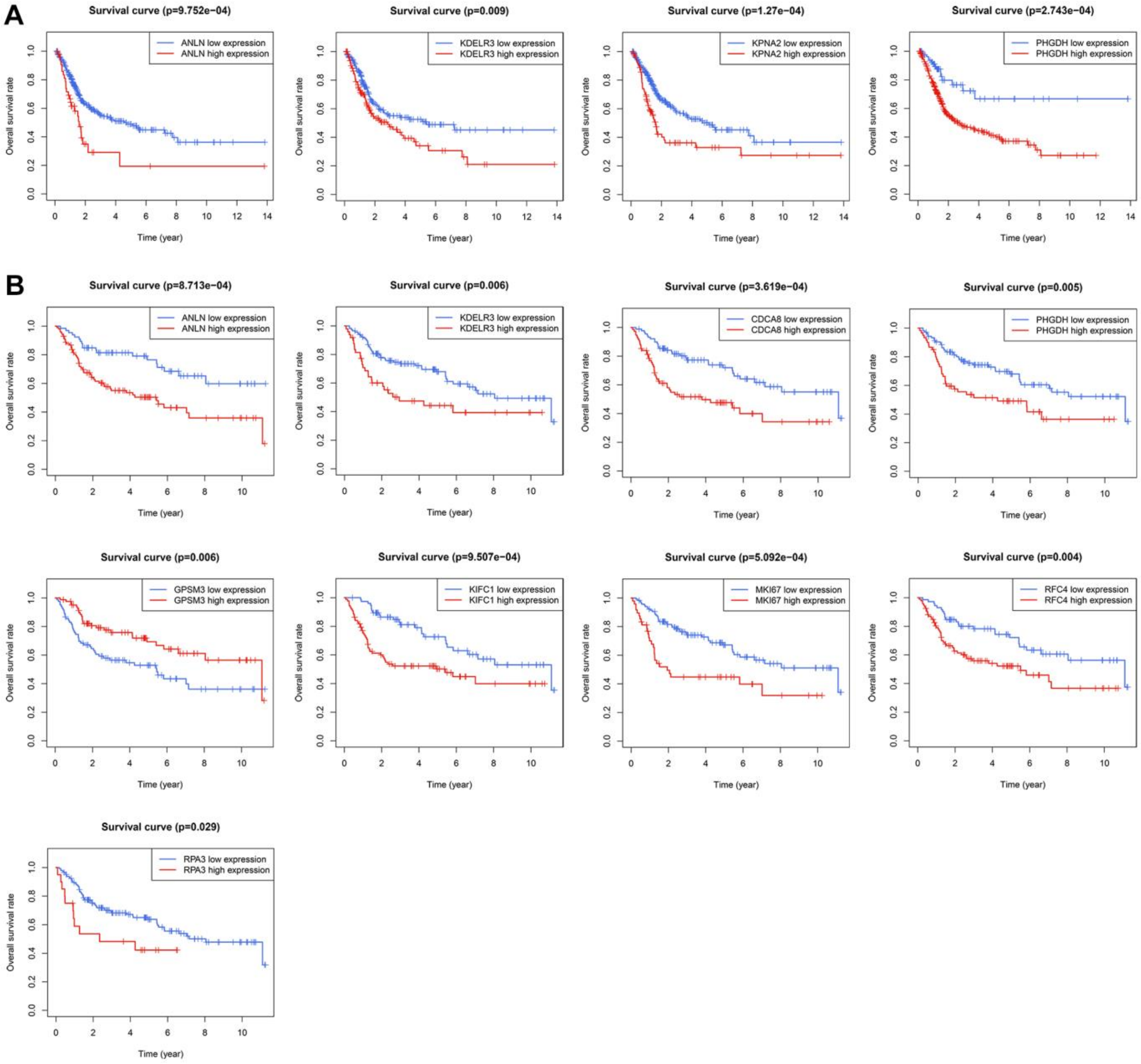

Supplementary Figure 4. Kaplan-Meier survival analysis of the 13 genes in TCGA (**A**) and GSE13507 (**B**) datasets. The optimal cut-off was determined through X-tile.

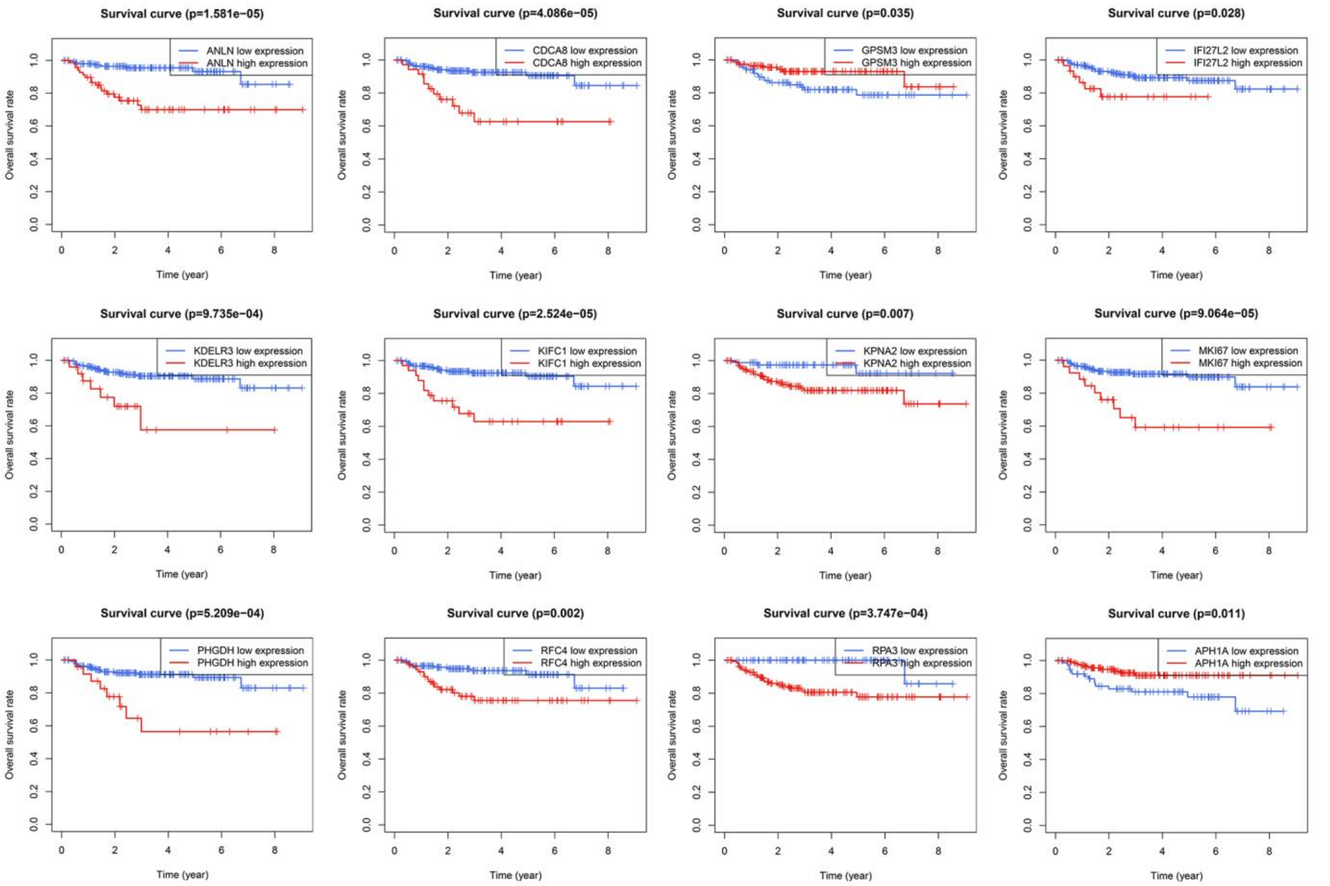

Supplementary Figure 5. Kaplan-Meier survival analysis of the 13 genes in GSE32894 cohort. X-tile was used for detecting the optimal cut-off value.

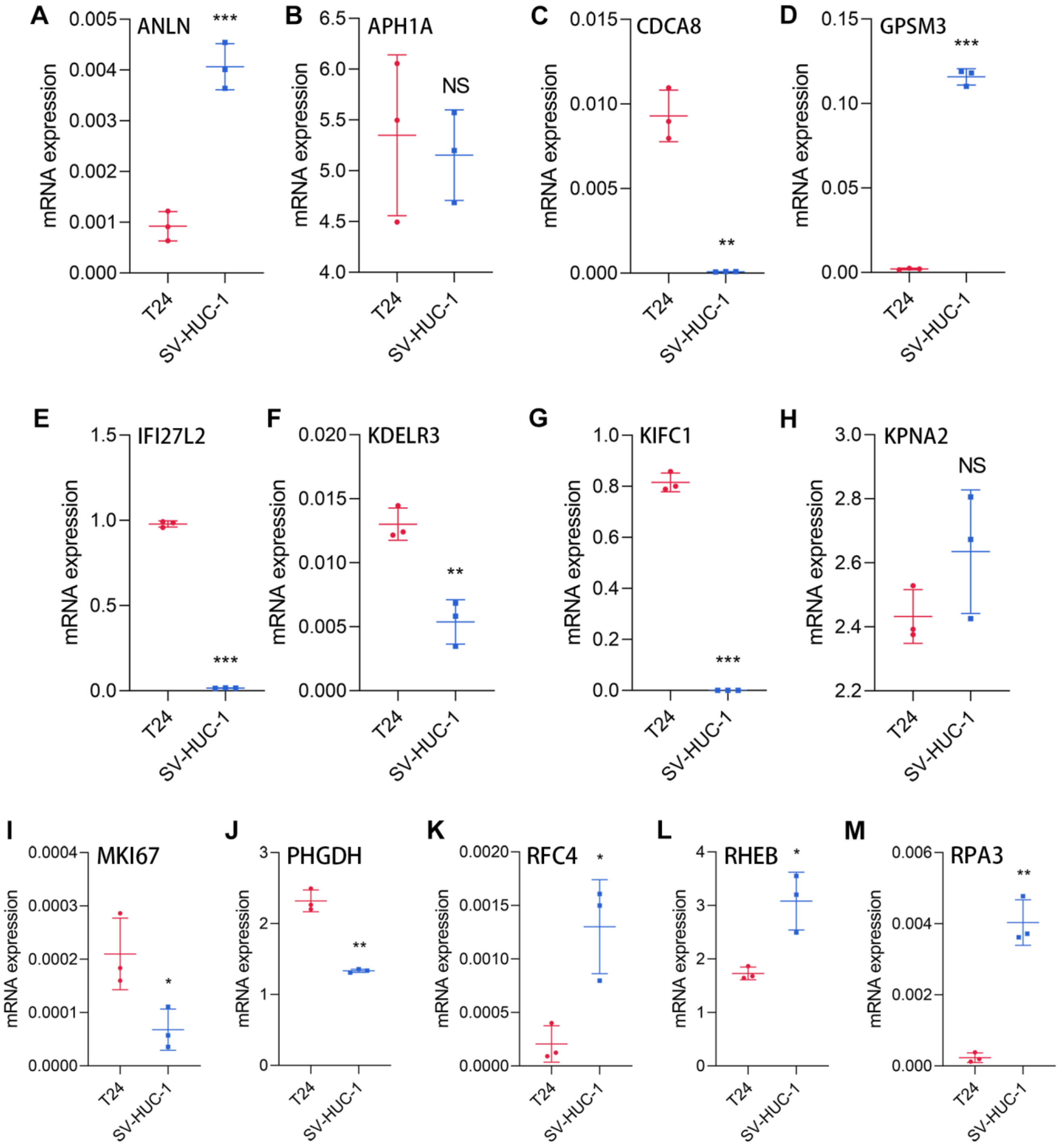

Supplementary Figure 6. The expression values of the 13 HRS genes, including ANLN (**A**), APH1A (**B**), CDCA8 (**C**), GPSM3 (**D**), IFI27L2 (**E**), KDELR3 (**F**), KIFC1 (**G**), KPNA2 (**H**), MKI67 (**I**), PHGDH (**J**), RFC4 (**K**), RHEB (**L**), and RPA3 (**M**), in T24 cells and SV-HUC-1 cells via RT-qPCR detection. *$p < 0.05$; **$p < 0.01$; ***$p < 0.001$.
