# Supplementary material for: Development and validation of an intra-tumor heterogeneity-related signature to predict prognosis of bladder cancer: a study based on single-cell RNA-seq
Source: Aging (Albany NY). 2021 Aug 2;13(15):19415–41. doi: 10.18632/aging.203353 (PMC8386527; doi:10.18632/aging.203353)
Supplement: Supplementary Tables 5, 9-13 [file aging-13-203353-s006.pdf]

SUPPLEMENTARY TABLES

Supplementary Table 5. The details of the established model constructed via multivariate Cox regression.

Id	coef	HR	HR.95L	HR.95H	*p* value
KDELR3_RFC4	0.59906044	1.820407615	1.284993084	2.578911845	0.000749008
GPSM3_IFI27L2	–0.437820457	0.645441657	0.452983804	0.919668494	0.015371371
ANLN_MKI67	0.374716571	1.454579086	1.0085688	2.097824479	0.0448955
ANLN_CDCA8	0.589653534	1.803363503	1.199952669	2.710206833	0.004554417
ANLN_KIFC1	0.343999231	1.410577551	0.946384138	2.102453906	0.091154079
KPNA2_APH1A	0.501950219	1.651939776	1.164418355	2.343577815	0.004907379
RPA3_PHGDH	–0.623979584	0.535807896	0.324557473	0.884558593	0.01470563
RHEB_PHGDH	–0.41459758	0.660606069	0.464862245	0.938773546	0.020758688

Supplementary Table 9. The *p* values of the 13 genes to OS via Kaplan-Meier survival analysis with log-rank test in TCGA cohort.

Gene	*p* value
KDELR3	0.0089878
GPSM3	0.0552757
ANLN	0.0009752
KPNA2	0.000127
RPA3	0.1100399
RHEB	0.3161838
RFC4	0.3074479
IFI27L2	0.1198286
MKI67	0.1708345
CDCA8	0.2304972
KIFC1	0.5828702
APH1A	0.0906864
PHGDH	0.0002743

Supplementary Table 10. The *p* values of the 13 genes to OS via Kaplan-Meier survival analysis with log-rank test in GSE13507 cohort.

Gene	*p* value
KDELR3	0.0058833
GPSM3	0.0062059
ANLN	0.0008713
KPNA2	0.1358367
RPA3	0.0288977
RHEB	0.1812605
RFC4	0.0043104
MKI67	0.0005092
CDCA8	0.0003619
KIFC1	0.0009507
APH1A	0.1464289

PHGDH	0.0050507
IFI27L2	0.0824967

Supplementary Table 11. The *p* values of the 13 genes to OS via Kaplan-Meier survival analysis with log-rank test in GSE32894 cohort.

Gene	*p* value
KDELR3	0.000973
GPSM3	0.035384
ANLN	1.58E-05
KPNA2	0.00709
RPA3	0.000375
RHEB	0.331377
RFC4	0.002442
MKI67	9.06E-05
CDCA8	4.09E-05
KIFC1	2.52E-05
APH1A	0.011216
PHGDH	0.000521
IFI27L2	0.028095

Supplementary Table 12. The primers utilized in present study.

Id	Forward primer (5′ –> 3′)	Reverse primer (5′ –> 3′)
KDELR3	TCCCAGTCATTGGCCTTTCC	CCAGTTAGCCAGGTAGAGTGC
GPSM3	AGGAGTTTTTCCCAGTCTCAGT	TTCTCTTCCCACCCAAACAGC
ANLN	TGCCAGGCGAGAGAATCTTC	CGCTTAGCATGAGTCATAGACCT
KPNA2	CTGCCCGTCTTCACAGATTCA	GCGGAGAAGTAGCATCATCAGG
RPA3	AGCTCAATTCATCGACAAGCC	TCTTCATCAAGGGGTTCCATCA
RHEB	TTGTGGACTCCTACGATCCAA	GGCTGTGTCTACAAGTTGAAGAT
RFC4	CCGCTGACCAAGGATCGAG	AGGGAACGGGTTTGGCTTTC
IFI27L2	CCACATCATCCAACATCCTCC	TCATCTTCTTTAGCCTCGGGTT
MKI67	ACGCCTGGTTACTATCAAAAGG	CAGACCCATTTACTTGTGTTGGA
CDCA8	GAAGGGCAGTAGTCGGGTG	TCACGGTCGAAGTCTTTCAGA
KIFC1	GGTGCAACGACCAAAATTACC	GGGTCCTGTCTTCTTGGAAAC
APH1A	TTTTTCGGCTGCACTTTCGTC	TGCGACCAGGATGATAACGC
PHGDH	CTGCGGAAAGTGCTCATCAGT	TGGCAGAGCGAACAATAAGGC

Supplementary Table 13. The results of GSVA analysis.

Id	logFC	AveExpr	*t*	*p* value	adj. *P*. Val	B
HALLMARK_MTORC1_SIGNALING	0.091526202	−0.04214319	4.418777	1.28E-05	0.0006389	2.8557429
HALLMARK_OXIDATIVE_PHOSPHORYLATION	−0.102512372	−0.04601685	–4.206509	3.20E-05	0.0015677	2.0000334
HALLMARK_MITOTIC_SPINDLE	0.090202864	−0.04325266	4.1063592	4.87E-05	0.0023374	1.6096638
HALLMARK_DNA_REPAIR	−0.0680874	−0.04970153	–3.971025	8.48E-05	0.0039839	1.0958871
HALLMARK_PEROXISOME	−0.059440425	−0.03764865	–3.824692	0.0001517	0.006978	0.5582572
HALLMARK_G2M_CHECKPOINT	0.12383884	−0.02058956	3.6655883	0.0002798	0.012592	−0.005001

HALLMARK_EPITHELIAL_MESENCHYMAL_TRANSITION	0.136256246	−0.01514403	3.5184996	0.0004835	0.0212741	−0.50582
HALLMARK_E2F_TARGETS	0.11558866	−0.01157635	3.0847107	0.002178	0.0936557	−1.869989
HALLMARK_FATTY_ACID_METABOLISM	−0.045102033	−0.04568334	–2.986091	0.0029983	0.1259277	−2.156347
HALLMARK_PROTEIN_SECRETION	0.068457724	−0.03805288	2.9706073	0.0031502	0.129157	−2.200499
HALLMARK_ANGIOGENESIS	0.092499901	−0.025644	2.918166	0.0037184	0.1487362	−2.348405
HALLMARK_ANDROGEN_RESPONSE	0.054163091	−0.04380175	2.8860864	0.0041106	0.1603138	−2.437639
HALLMARK_HYPOXIA	0.057318158	−0.0384402	2.8665723	0.0043673	0.165956	−2.491458
HALLMARK_BILE_ACID_METABOLISM	−0.043584756	−0.04440084	–2.837239	0.0047806	0.1768806	−2.571699
HALLMARK_ADIPOGENESIS	−0.038230578	−0.04633641	–2.596801	0.0097542	0.3511514	−3.199459
HALLMARK_UV_RESPONSE_DN	0.057263757	−0.04566087	2.5444044	0.011319	0.3961639	−3.329144
HALLMARK_UNFOLDED_PROTEIN_RESPONSE	0.043122271	−0.04807679	2.476172	0.0136901	0.465464	−3.494188
HALLMARK_SPERMATOGENESIS	0.035904577	−0.02105281	2.4301759	0.0155276	0.5124106	−3.602991
HALLMARK_TGF_BETA_SIGNALING	0.055378448	−0.04224624	2.3295063	0.020326	0.6504325	−3.834211
HALLMARK_REACTIVE_OXYGEN_SPECIES_PATHWAY	−0.04290982	−0.05314658	–2.27996	0.0231323	0.7170998	−3.944516
HALLMARK_TNFA_SIGNALING_VIA_NFKB	0.070965528	−0.03196946	2.2615103	0.0242604	0.7278121	−3.985
HALLMARK_COAGULATION	0.056094838	−0.01821846	2.2064716	0.0279148	0.8095302	−4.103868
HALLMARK_HEDGEHOG_SIGNALING	0.052376405	−0.04787308	2.205812	0.0279614	0.8095302	−4.105275
HALLMARK_GLYCOLYSIS	0.0313427	−0.04681433	2.161573	0.0312398	0.8434745	−4.198719
HALLMARK_INFLAMMATORY_RESPONSE	0.069240648	−0.02762496	2.0932041	0.0369561	0.9608593	−4.339494
HALLMARK_MYC_TARGETS_V1	0.05896169	−0.03787073	2.0074322	0.0453714	1	−4.509842
HALLMARK_APICAL_JUNCTION	0.043373326	−0.04002215	2.006346	0.0454876	1	−4.511954
HALLMARK_XENOBIOTIC_METABOLISM	−0.027357847	−0.03182934	–1.875082	0.061505	1	−4.758987
HALLMARK_COMPLEMENT	0.043843668	−0.03093177	1.6110832	0.1079452	1	−5.206029
HALLMARK_KRAS_SIGNALING_UP	0.039222297	−0.03820804	1.5973272	0.1109772	1	−5.227493
HALLMARK_MYC_TARGETS_V2	0.047231895	−0.03622899	1.4396179	0.1507525	1	−5.460575
HALLMARK_IL6_JAK_STAT3_SIGNALING	0.044389386	−0.02740203	1.35617	0.1758051	1	−5.574214
HALLMARK_APOPTOSIS	0.022787978	−0.03955432	1.1226794	0.2622427	1	−5.856423
HALLMARK_NOTCH_SIGNALING	−0.020422791	−0.04917705	–1.120955	0.2629753	1	−5.858312
HALLMARK_IL2_STAT5_SIGNALING	0.025344043	−0.04030152	1.0703473	0.2851042	1	−5.912431
HALLMARK_UV_RESPONSE_UP	−0.012238878	−0.04667981	–0.906738	0.3650877	1	−6.070377
HALLMARK_P53_PATHWAY	−0.012958715	−0.04582705	–0.739255	0.460183	1	−6.205088
HALLMARK_PANCREAS_BETA_CELLS	0.011969347	0.002796107	0.607188	0.5440684	1	−6.292031
HALLMARK_HEME_METABOLISM	0.0066419	−0.04233163	0.5188347	0.604161	1	−6.340692
HALLMARK_ALLOGRAFT_REJECTION	0.013818118	−0.02391175	0.3724455	0.7097571	1	−6.404527
HALLMARK_KRAS_SIGNALING_DN	−0.005060721	−0.0324103	–0.366763	0.7139888	1	−6.406582
HALLMARK_INTERFERON_GAMMA_RESPONSE	0.01207391	−0.02766363	0.3239049	0.7461782	1	−6.421068
HALLMARK_CHOLESTEROL_HOMEOSTASIS	0.005372206	−0.04847371	0.3000273	0.7643112	1	−6.428358
HALLMARK_ESTROGEN_RESPONSE_EARLY	0.003545514	−0.04628076	0.2088272	0.8346888	1	−6.451071
HALLMARK_INTERFERON_ALPHA_RESPONSE	−0.007399147	−0.02553256	–0.18075	0.8566546	1	−6.456425
HALLMARK_APICAL_SURFACE	0.003052593	−0.04552941	0.161198	0.8720183	1	−6.459698
HALLMARK_ESTROGEN_RESPONSE_LATE	−0.002251102	−0.04602582	–0.1469	0.8832846	1	−6.461854
HALLMARK_MYOGENESIS	0.002295383	−0.03740793	0.0912882	0.927309	1	−6.468338
HALLMARK_WNT_BETA_CATENIN_SIGNALING	0.001386947	−0.05009891	0.0732182	0.9416688	1	−6.469793
HALLMARK_PI3K_AKT_MTOR_SIGNALING	0.000522011	−0.04980514	0.0321418	0.9743749	1	−6.471911
